# Housefly (*Musca domestica* L.) associated microbiota across different life stages

**DOI:** 10.1038/s41598-020-64704-y

**Published:** 2020-05-12

**Authors:** Nadieh de Jonge, Thomas Yssing Michaelsen, Rasmus Ejbye-Ernst, Anne Jensen, Majken Elley Nielsen, Simon Bahrndorff, Jeppe Lund Nielsen

**Affiliations:** Department of Chemistry and Bioscience, Aalborg University, DK-9220 Aalborg East, Denmark

**Keywords:** Non-model organisms, Microbiome

## Abstract

The housefly (*Musca domestica* L.) lives in close association with its microbiota and its symbionts are suggested to have pivotal roles in processes such as metabolism and immune response, but it is unclear how the profound physiological changes during ontogeny affect the housefly’s associated microbiota and their metabolic capabilities. The present study applies 16S rRNA gene amplicon sequencing to investigate the development of the host-associated microbiota during ontogeny. The potential for microbiota transfer between developmental stages, and the metabolic potential of these microbiota were evaluated. Representatives of *Firmicutes* were observed as early colonisers during the larval stages, followed by colonisation by organisms affiliating with *Proteobacteria* and *Bacteroidetes* as the flies matured into adults. Microbiota observed across all the developmental stages included *Lactococcus*, *Lactobacillus* and *Enterococcus*, while *Weissella* and *Chishuiella* were associated with newly hatched larvae and adults, respectively. Predictive metabolic profiling of the identified microorganisms further suggested that the microbiota and their functional profile mature alongside their host and putative host-microbe relationships are established at different stages of development. The predicted metabolic capability of the microbiota developed from primarily simple processes including carbohydrate and nucleotide metabolisms, to more complex metabolic pathways including amino acid metabolisms and processes related to signal transduction.

## Introduction

The symbiotic relationship between host and its microbiome is universal and has significant impact on numerous phenotypical traits, including host fitness, health, immune response, fertility and even behaviour as shown in many different types of organisms including insects^1–3^. Host microbiota composition and host fitness generally have a dynamic relationship that is continuously affected by factors such as environmental changes, age and developmental stages of the host life cycle. Important factors for these interactions are based on physical parameters originating from the habitat which eventually influence the intestinal environment^1,4^, thus requiring the development of a host microbiota composition that is able to adapt to this continuous change.

Host associated microbiota is often characterised by strong symbiotic relationships^5^. The most well-described example of host associated microorganisms in insects is the intracellular endosymbiont *Wolbachia*, which has been found in wide range of arthropods^6^, including houseflies^7^ and wasps^8^. This bacterium has been linked to significant alterations in host biology, such as feminisation and sperm-egg incompatibility, and may have a mutualistic or parasitic relationship with its host depending on species^6^. Another well-studied example of a host-microbe encompasses the interaction between the ecology of the gut and associated endosymbionts in termite species, where specialised microbiota are required for the survival of subterranean termites in order to digest lignocellulosic materials^9^. Furthermore, microbial protection of the heritable endosymbiotic organism *Hamiltonella defensa* against predatory wasps in pea aphids (*Acyrthosiphon pisum*) has also been described^10^. Moreover, representatives of the *Enterobacteriaceae* present in the gut microbiota of pine weevils (*Hylobius abietis*) have been shown to be responsible for the degradation of diterpenes present in the bark of conifer trees, and the lack of these microbes has a negative effect on host fitness and reproduction^11^.

Continuous development of the microbiota composition throughout the host life cycle has been observed in several species of insects, including malaria mosquitoes (*Anopheles gambiae*)^12^, black soldier flies (*Hermetia illucens* (L.))^13^, and cotton leaf worms (*Spodoptera littoralis*)^14^. These studies showed that the development of the microbiota could be linked to the physiological changes of the host, as well as dietary changes throughout the host life cycle, suggesting that the functional profile of the microbiota develops alongside the host. The most widely observed transfer and exchange of microbiota in insects takes place through vertical transmission following hormone induced evolution of the host, such as the maternal transfer of *Buchnera aphidicola* across generations in pea aphids (*Acyrthosiphon pisum*)^15^. Reproductive transfer schemes may be especially relevant for mutualistic bacteria associated with hosts. For example, female houseflies have been shown to deposit an endosymbiotic bacterium (*Klebsiella oxytoca*) in order to prevent further oviposition at the site of newly laid eggs^16^. Changes in the host associated microbiota can also occur through horizontal transfer of microbes through exchange with the environment, as well as between insects; this has previously been observed in species with a social behaviours such as ants, bees and termites^2^.

Houseflies (*Musca domestica L*.) are members of the insect family *Muscidae*, which contains a number of species considered as vectors of disease. They are considered filth flies due to their association with organic substrates such as household waste and manures^17^. Due to their close association with humans, these insects and their microbiome are of interest for detailed study. Houseflies have an expected life span of approximately 30 days, during which they go through four distinct developmental stages; egg, larvae (S1, S2 and S3), (pre)pupa, and adult^18^. Life cycle and host dependent microbiota have previously been reported in related organisms such as black soldier flies^13^, and it can thus be hypothesised that houseflies are subject to dependent microbiota with specific functionalities. This is supported by a previous study in which differences in microbiota associated with housefly larvae and adult samples were observed^19^.

A number of studies have investigated the microbiota of the housefly. A recent next generation sequencing (NGS) study of the microbiota associated with wild adult houseflies showed that houseflies represent a highly complex microbial ecosystem, likely reflecting the lifestyle of the individual within its habitat^20^,^21^. The microbiota associated with housefly larvae, pupae and adults has also been investigated using denaturing gradient gel electrophoresis (DGGE), with focus on antibiotic resistant bacteria^22^. Housefly larvae feeding on wheat bran have been shown to exchange gut microbiota with the environment, supporting that the life style at different life stages of the housefly influences the composition of their microbiota^23^. Furthermore, dissection of the gut in larvae and adult flies revealed the presence of a growth stage dependent microbiota, as well as potential symbionts that persist through morphological transformation into adult flies^19,24^. These observations support the presence of microbiota with a functional role which depends on and develops alongside the physiological state of the host. However, the knowledge regarding the microbial community associated to all housefly stages of development, transfer of potential symbionts between stages of development and generations, as well as their potential function is still lacking.

The present study aims to deepen the knowledge relating to the microbiota of the housefly during ontogeny. Amplicon sequencing of the 16S rRNA gene was applied to the different developmental stages of laboratory reared houseflies in order to establish the structural composition of the microbiota associated with the different stages of life. Differences in the microbial communities identified between developmental stages, and differentiation in body compartments and across genders of adult flies were investigated to explore transfer and development of microbial communities across morphological stages. Finally, the metabolic potential of the microbiota associated to the different developmental stages was assessed to reveal putative host-microbe interactions, mechanisms and microbiota functionalities.

## Results

A total number of 37 samples were subjected to 16S rRNA gene amplicon sequencing, consisting of 32 tissue samples and 5 samples of the rearing media (Fig. S1). High quality sequences were obtained for triplicate measurements of 25 pooled fly specimens (n = 14) and media samples of all developmental stages except the egg stage, where only one sample out of three was successfully sequenced. The individually analysed adult flies yielded 10 samples from the F1 generation (3 females and 7 males) and one sample from F2 adults (female). A total of 945,095 sequences were generated with an average of 26,563 ± 10,419 sequences per sample (Fig. S1). The lowest number of reads in a sample was 3,894 (medium of S2 larvae), however a rarefaction curve (Fig. S2) showed that all curves approximated a horizontal asymptote, therefore all samples entered downstream analysis. Biological triplicate measurements of the developmental stage samples were highly similar in composition as investigated by hierarchical clustering of Bray-Curtis distances between samples (Fig. S3), and tested by ANOSIM (p = 0.001, R^2^ = 0.4363). The dendrogram generated based on the clustering also showed that with the exception of larval stage S1, the media samples of larval stages S2 and S3 as well as the (pre)pupa stages clustered away from the respective tissue samples.

### Development of housefly microbiota during the host life cycle

A cumulative trend was observed with regards to microbial community richness throughout the sampled developmental stages (Table 1); observed microbial diversity increased steadily from approximately 40 OTUs per sample at the S1 larval stage until it stabilised around 200 OTUs per specimen at the adult stage of the F2 generation (Table 1). Pairwise Wilcoxon rank sum testing of the microbial community richness between the life stages where three replications were present did not reveal significant differences. The microbial evenness scores ranged from 0.1 to 3.2 on the Shannon-Weaver index (Table 1). An unexpectedly high diversity (1,229 OTUs) was measured for the egg sample. Microbial communities observed in F2 adult flies and body parts were marginally more diverse than that of the F1 adult flies (p = 0.097). No significant differences were observed between male and female flies in terms of richness (p = 0.27).

**Table 1:** Alpha diversity measurements on the observed number of OTUs (97%) identified in the developmental stages of *M. domestica* (25 specimens per sample for all life stages except the individually analysed adult specimens), n indicates the number of biological replications (mean ± sd).

Developmental stage	Observed OTUs	Shannon index
Adult F1 (n = 10)	107 ± 26	1.5 ± 0.6
Egg (n = 1)	1,229	5.7
Larvae S1 (n = 3)	37 ± 19	0.1 ± 0.0
Larvae S2 (n = 2)	87 ± 25	1.2 ± 0.1
Larvae S3 (n = 3)	124 ± 12	2.2 ± 0.1
Pre-pupa (n = 3)	176 ± 43	3.2 ± 0.5
Pupa (n = 3)	144 ± 47	1.7 ± 0.8
Adult F2 (n = 1)	227	3.2

**Figure 1:**
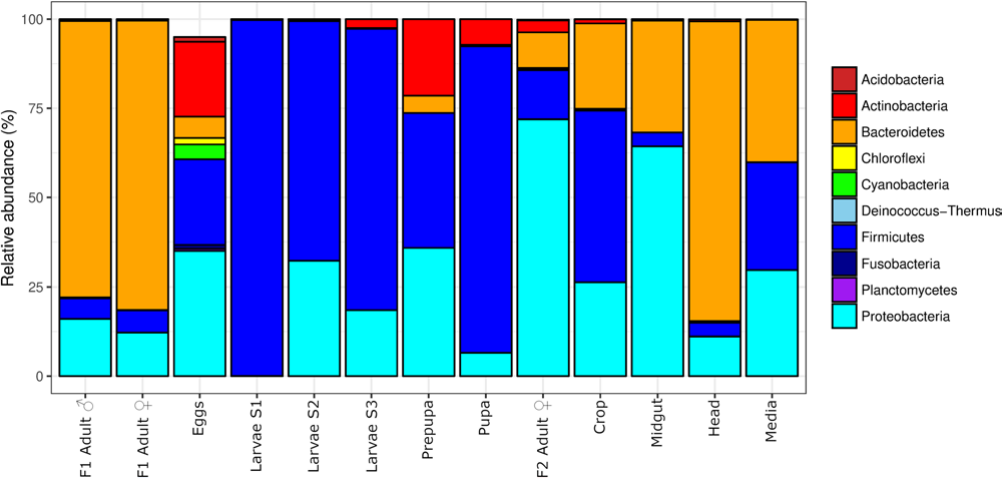
Life cycle microbial community structure. Microbial community structure at phylum level across sampled developmental stages and body parts, shown as a stacked barplot. The ten most abundant phyla are displayed. Each sample represents a pool of 25 individuals, except for the individually analysed adult specimens.

At phylum level, the most abundant contributor to the microbiota observed in the F1 adult flies were representatives of *Bacteroidetes* (Fig. 1), while the most abundantly detected phylum in the microbiota across the developmental stages of the F2 generation was *Firmicutes*. Starting from larval stage S2, the *Proteobacteria* became an abundant contributor to the microbiota, and from the pre-pupa stage, OTUs representing *Actinobacteria* were also abundantly detected. The F2 adult samples (single fly and body parts) contained microbiota that were made up of a mixture of *Bacteroidetes*, *Firmicutes*, and *Proteobacteria*.

**Figure 2:**
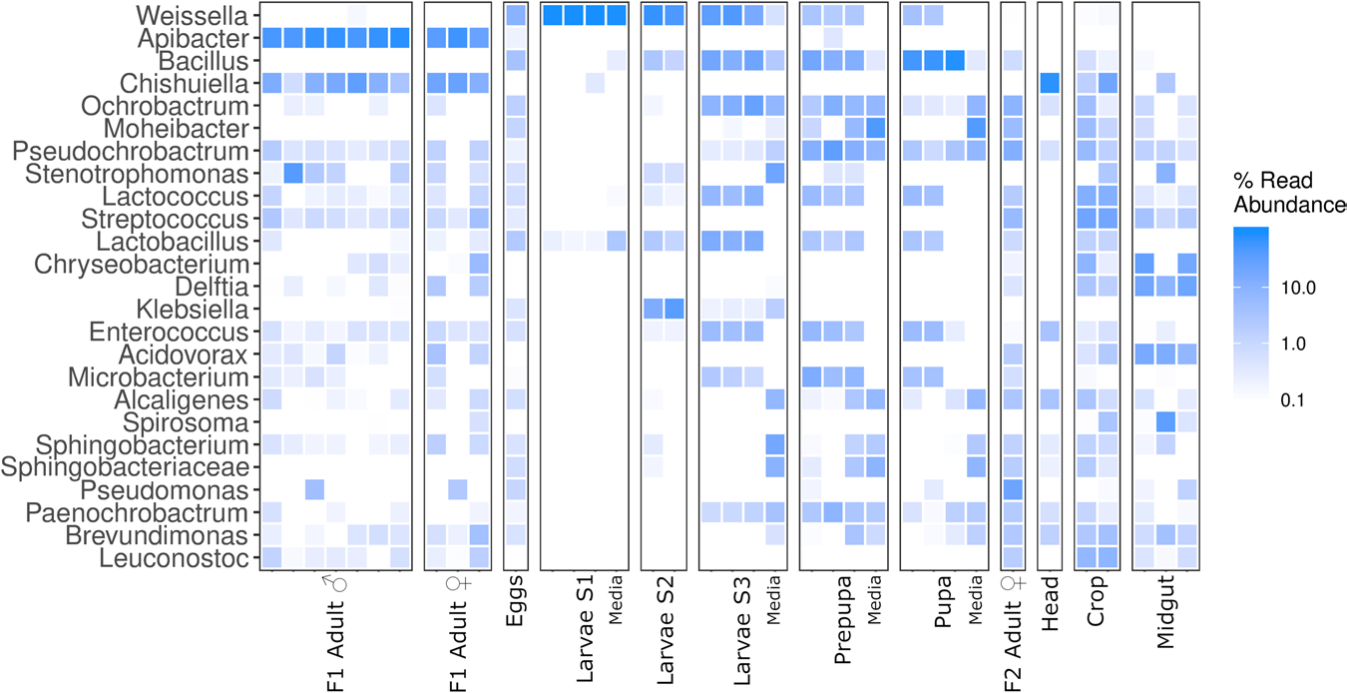
Microbial community composition during *M. domestica* life cycle. Heatmap representing the most abundantly observed OTUs, grouped to and displayed as the 25 most abundant genera identified across sampled developmental stages, media and body parts. Each sample represents a pool of 25 individuals, except for the individually analysed adult specimens.

At high taxonomic resolution (genus level), five genera were observed abundantly in the majority of developmental stages, and both generations (F1 and F2) of flies; *Pseudochrobactrum*, *Lactococcus, Lactobacillus, Enterococcus* and *Paenochrobactrum* (Fig. 2). Newly hatched larvae (S1) were predominantly colonised by *Weissella* with up to 99% of total read abundance and *Lactobacillus* at low read abundances. During the development of the larval stages, the microbiota diversified, and gained abundant presences of *Stenotrophomonas, Bacillus* and *Lactococcus* during larval stage 2 (S2). Subsequently, members of the order *Rhizobiales* (*Ochrobactrum, Pseudochrobactrum* and *Paenochrobactrum*) and other lactic acid bacteria (LAB) such as *Lactococcus*, emerged during larval stage 3 (S3). *Klebsiella* was observed abundantly and exclusively during the larval stages S2 and S3, and *Microbacterium* and *Enterococcus* were almost exclusively found during larval stage S3 and (pre-)pupa stages. Other genera exclusively present during developmental stages (egg to pupa stage) of the fly included *Weissella* and *Bacillus*. Members of the *Betaproteobacteria* (*Acidovorax* and *Delftia)*, and the genera *Streptococcus, Chishuiella, Chryseobacterium* and *Leuconostoc* were only detected in adult flies of either or both generations. The genus *Apibacter* represented the population with the highest read abundance (26.2–84.4% of total reads) in F1 adult flies, regardless of gender, and was only present in the F1 generation flies. The analysed media samples resembled the developmental stages cultivated on it during early development (larval stages S1 and S2), but differed from the microbiota of larval stages 3, pre-pupa and pupa stages. The presence of potential symbionts was investigated by examining the ubiquitous observed OTUs across the larval, pupal and adult life stages (Fig. S4). Two OTUs were found to be in all tested samples, and were affiliated to the genera *Weissella* and *Lactococcus*.

**Figure 3:**
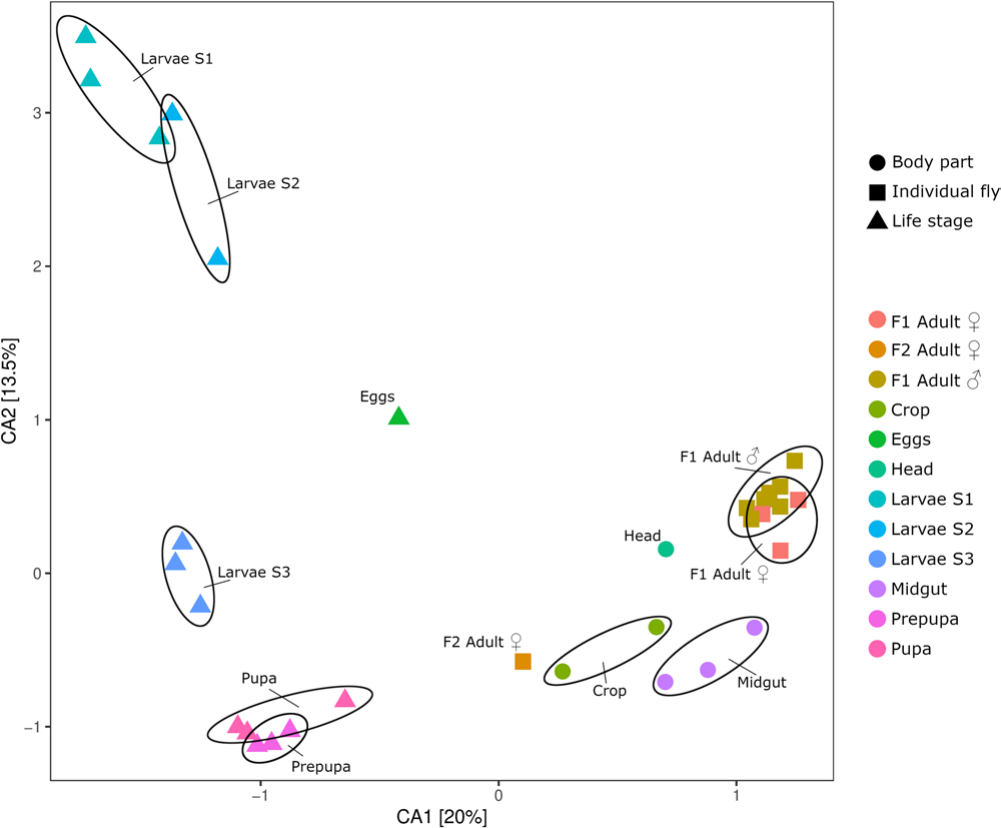
Life cycle microbial community evolution. Correspondence analysis (CA) on Hellinger transformed OTU abundance counts. Samples are coloured by sample type, and an ellipse is drawn around each individual developmental stage and group. Each sample represents a pool of 25 individuals, except for the individually analysed adult specimens.

A strong trend in the differences in the microbiome between life stages can be observed in Fig. 3, moving counter-clockwise from the samples representing larval stages S1 and S2, along the vertical axis towards larval stage S3, and the pre-pupa and pupa stage, which were clustered very closely together. The adult samples from both generations were clustered loosely together, away from the cluster containing the (pre-)pupa samples, and the adult body part samples from F2 were grouped together with the adult samples as well.

### Metabolic profiling suggests establishment and maturation of microbiome alongside host development

**Figure 4:**
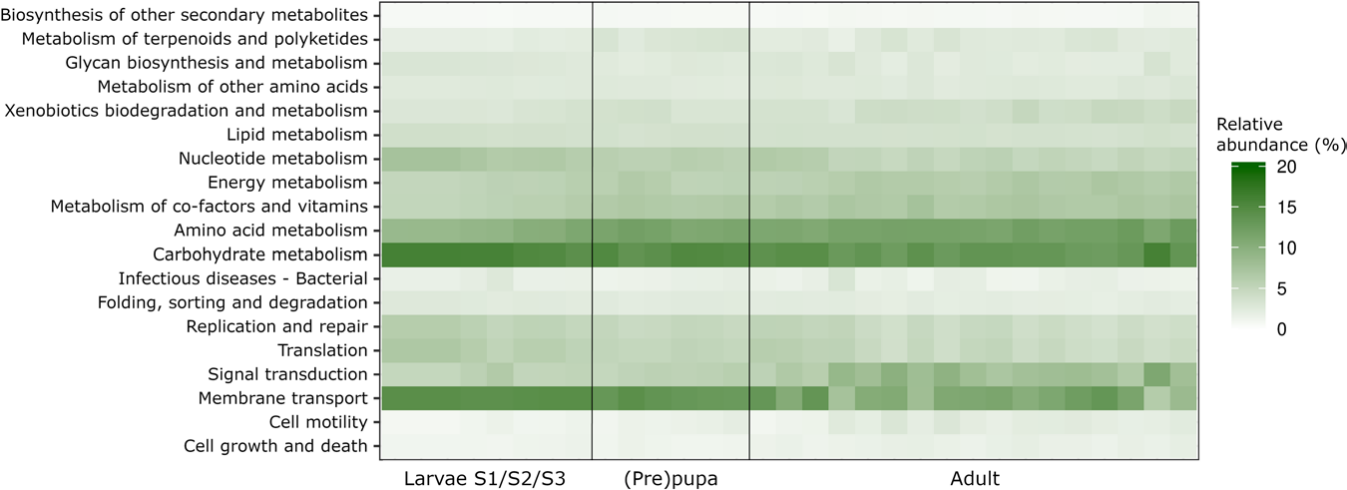
Metabolic potential of housefly associated microbiota. Heatmap of the relative distribution of abundant (at least 1% of total abundance in 1 sample) level 2 KEGG assignments by Tax4Fun. Samples are sorted according to their developmental stage. Each sample represents a pool of 25 individuals, except for the individually analysed adult specimens.

The development of a diverse housefly associated microbiota was also reflected by the metabolic profiles. Throughout the sampled developmental stages, a diversification and exchange of metabolic capabilities (KEGG level 2) was observed within the microbiota (Fig. 4). During the larval stages of development, the majority of predicted metabolic functions were associated with carbohydrate metabolism and membrane transport, as well as nucleotide metabolism. Pre-pupa and pupa associated microbiota showed an increase in relative abundance of amino acid metabolism, and metabolism of cofactors and vitamins, at the cost of nucleotide metabolism. The adult fly microbiota had a higher relative abundance of amino acid metabolism compared to larvae and (pre-)pupa stages, as well as increased abundance of signal transduction and xenobiotics biodegradation and metabolism.

### Housefly associated microbiota across gender and gut compartments

**Figure 5:**
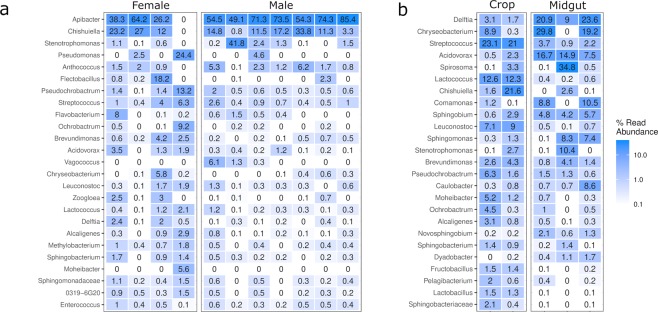
Microbial community composition in gut compartments and between genders. Heatmaps of the 25 most abundant genera observed in samples collected from whole adult fly microbiome samples, sorted by gender (**a**) and the crop and midgut segments of adult flies (**b**). Genera are displayed with the highest taxonomic resolution possible. Each sample represents a pool of 25 individuals, except for the individually analysed adult specimens.

Overall, the abundant microbiota observed between male and female adult flies (n = 11) (Fig. 5a) contained minor differences, and a similar pattern of variation between individual specimens was observed for both genders. Organisms that were observed abundantly for both genders included the genera *Apibacter, Chishiuella*, *Pseudochrobactrum, Anthococcus* and *Streptococcus*. Two genera were found to be gender-specific, namely *Flectobacillus* which was predominantly seen in females, and *Vagococcus* which was only detected in male adult flies.

The microbiota colonising the crop (n = 2) and midgut (n = 3) of adult flies were examined in detail (Fig. 5b). The majority of the identified organisms were observed to colonise both segments, although the abundances of the observed microbiota differed between segments. Representatives of the genera *Streptococcus, Lactococcus, Leuconostoc*, and *Chishuiella* were observed abundantly in the crop segment, whereas the midgut was most abundantly colonised by *Delftia, Chryseobacterium, Acidovorax, Comamonas, Spirosoma*, and *Sphingomonas*. A small number of low abundant microorganisms were primarily detected in either the crop or midgut; *Pelagibacterium*, *Fructobacillus* and *Lactobacillus* were mostly observed in the crop, while *Dyadobacter* and *Novosphingobium* were almost exclusively seen in the midgut samples.

## Discussion

Current knowledge about the housefly microbiota originates from studies carried out primarily in adult specimens^24^ and only very limited insight into the gender- or developmental stage specificity of microbiota exists. The majority of the observations made on the housefly microbiome derives from the context of host vector competence^22^. The current work presents a comprehensive look at the microbiota associated with the housefly throughout its life cycle and development. Furthermore, the gender-specificity of adult male and female microbiomes as well as two segments of the housefly gut were examined in detail. Metabolic profiling was performed to gain insight into the potential functional capabilities of the microbiota during different stages of life. The observed composition of the microbiota as well as its potential function correlated with the different life stages of the houseflies.

### Development of housefly microbiota across different life stages

An increasing diversity of microbiota was observed throughout the developmental cycle of the flies (n = 26). Richness increased from 40 OTUs during larval stage 1 to around 200 OTUs detected per sample at the adult stage (Table 1). The growing diversity of microbiota is likely a reflection of the lifestyle and physiological complexity of the individual developmental stages. This type of development has previously been hypothesized in black soldier flies^13^, and may be a reflection of changing host needs, and dependence on the interactions with its microbiota.

An unexpectedly high number of OTUs (1,229) was found in the egg sample and the egg microbiome had only little in common with the microbiota observed in subsequent developmental stages. Egg associated microbiota has previously been suggested to serve as nutrients for newly emerged larvae^25^, as well as a specialised role in relation to protection from larval cannibalism^16^. *Klebsiella* was observed as one of the abundant genera in eggs, which is in accordance with previous reports which proposed that it might affect oviposition^16^.

The changes in the microbial community composition through the different housefly developmental stages was characterised by a diversification of colonisation during the larval stage of development, followed by an exchange of new microbiota. A large shift in microbial community composition could be observed at low taxonomic level (Fig. 1), changing from highly abundant colonisation by *Bacteroidetes* in F1 adults to primarily *Firmicutes* during the developmental stages and finally a combination of *Bacteroidetes*, *Firmicutes* and *Proteobacteria* in F2 adult samples. This radical change in community structure has previously also been observed in malaria mosquitos (*Anopheles gambiae*)^12^, and has been attributed to developmental and environmental changes. It is likely that the profound physiological transformation as well as changing exposure to the environment also influenced the composition of the microbiota in the housefly. A previous study in housefly larvae also showed exchange of microbiota with wheat bran media related to feeding^23^, while third instar housefly larvae (S3) subsequently cease feeding prior to pupation^17^, thus reducing the contribution of the media to the microbiota composition. This is supported by the observation that the media samples collected from the early stages of fly development were similar to the larvae (Figure S3), while media samples from larvae stage 3 and (pre-) pupa stages were not.

The largest relative abundance of a single OTU detected in the present study was identified to be a representative affiliated with *Apibacter*, an organism reported to be an endosymbiont in honeybees^26^. Other abundantly observed organisms included representatives of the genera *Weissella*, *Bacillus*, *Lactococcus*, *Lactobacillus* and *Pseudochrobactrum*, which occurred in differing stages of life, and were also found to be part of the ubiquitously observed groups in the larvae, pupae and adult samples (Fig. S4). These organisms have previously been observed in wild adult specimens of houseflies and other insects^20,27^. Another abundant organism represented *Orchrobactrum*, which has previously been proposed as an endosymbiont in nematode species^28^. Furthermore, colonisation by *Lactobacillus* and *Enterococcus* is also in line with previously observed microbial communities associated with organisms within the insect order Diptera^29^. Occurrence of the endosymbiont *Wolbachia* has previously been reported in wild houseflies^30^, and can represent a large proportion of the detected microbiota, as previously reported in a study in fruitflies (*Drosophila melanogaster*)^31^. However, *Wolbachia* was not detected in the present study.

The continued differentiation of the observed microbiota during the housefly life cycle and relative similarities between the overall microbiota of adult specimens of the F1 and the analysed female from the F2 generation as well as the F2 gut compartments, suggest that host associated microorganisms may be passed on through generations in houseflies. Limited vertical transmission of microbiota in insects has previously been observed for endosymbionts such as *Buchnera aphidicola*^15^. Furthermore, other potential modes of microbiota transmission have also been described for the reproductive tissues in arthropods^32^. Based on the progressive evolution of housefly microbiota throughout the developmental stages of the housefly, it may serve as a useful model organism for host-microbe interaction studies.

Few differences were observed between male and female microbiota, and it is likely that microbial community variation between individuals obscured gender-specific differences due to the low number of replications and usage of individual flies for this analysis. An example of housefly gender specific organisms is the symbiotic *Klebsiella oxytoca*, which is associated to newly laid housefly eggs, where it is deposited by the female, and has a role in oviposition as well as protection against potential pathogens^16,33^. No additional gender specific microbiota have been described for the housefly, however it can be hypothesised that these organisms primarily have roles related to reproductive processes. A previous study in two species of mosquitos (*Anopheles*) focused on microbiota associated to the reproductive organs also found a high degree of similarities between the microbial composition of male and female specimens, with a low number of microorganisms associated to either males or females^34^. The study further suggested that breeding location, desired environmental traits, reproductive status and interactions between different populations influence the ability to detect gender specific microbes. Large variation between individuals has previously been observed in studies on wild housefly populations^20^. A similar observation was made for the samples from individual body compartments (head, crop, midgut). However, a greater representation of lactic acid bacteria (*Lactobacillus*, *Lactococcus, Leuconostoc*) was observed in the crop compared to the midgut. This group of bacteria is known for performing carbohydrate fermentation of complex molecules to lactic acid^35^, and the presence of these microorganisms is in line with the function of the crop segment.

### Metabolic potential of housefly microbiota across life stages

To assess the potential functionality of the resident microbiota during the different developmental stages of the housefly, the metabolic profiling was performed using Tax4Fun. Predictive metabolic profiling is a method where the observed 16S rRNA gene amplicons are used to infer the potential function of a given microbial community, with prominent examples being PICRUSt^36^ and Tax4Fun^37^. Due to their dependence on existing (and often out-dated) databases, as well as their partial reliance on the taxonomic relationships between OTUs, results should be interpreted carefully. However, predictive metabolomic data based on 16S rRNA amplicon data of diverse datasets has been shown to recover metabolic trends in >50% of metabolites investigated^38^. This suggests that while highly theoretical, the predicted data may be a useful tool for the development of hypotheses and experimental designs.

Changes to the functional profiles of the microbial community of each developmental stage were observed. This suggests that the functionality of the microbiota evolves together with the host. During the early stages of development, the majority of metabolic relative abundance is assigned to carbohydrate metabolism, while at the adult stage the KEGG pathways (level 2) for amino acid and other energy metabolisms are also abundantly represented. This can be explained by the relative high dependence of carbohydrates as the dominant nutrient source during larval stages, while the more complex milk powder is important for the adult flies^27^. A similar shift in microbiota and predicted functionality thereof has previously also been reported in cotton leafworms (*Spodoptera littoralis*)^14^. Overall these results support the hypothesis that the housefly microbiota develops alongside the host during maturation.

In conclusion, this work presents novel insight into the development of housefly associated microbiota throughout its cycle of life. Continuous development of microbiota composition was observed for all stages of life, showing an increasing diversity of microorganisms up to the adult stage. A number of known insect endosymbionts were observed abundantly, including *Apibacter*, *Spirosoma* and *Klebsiella*. Several organisms were present throughout most of the life cycle and carried over through generations, while others appeared to be specific to developmental stages and/or compartments of the body. Prediction of the metabolic capabilities suggested that microbiota have different roles during host development and their functions diversify alongside the composition of the microbiota, dependent on host lifestyle. Carbohydrate and nucleotide metabolism were the most abundant functional pathways during the larval stages, other metabolic routes including amino acid metabolism, degradation of xenobiotics and signal transduction became abundant during the pupae and adult developmental stages.

## Methods

### Sample collection and cultivation

Adult houseflies were captured in September 2015 at Aalborg Zoo, Aalborg, Denmark (57°02′13.4″N 9°53′53.0″E) using aerial insect nets and transported to the lab in plastic net cages (Bugdorm, Taiwan). Members of species *M. domestica* were identified based on morphological features^18^. The collected specimens of *M. domestica* were reared as previously described^39^, in a temperature chamber at 25 ± 1 °C with a 12 h:12 h light/dark cycle with continuous access to water and food (white sugar and icing sugar) in addition to milk protein in order to facilitate oviposition^40^. As the present study was focused on the comparison of microbiota associated with different life stages living in the same environment, no strict steps were taken to sterilise the cultivation materials^22^. Newly laid eggs (F1) were transferred to a new container with fresh larval medium, aerated by daily stirring. Pupae from this generation were separated from the medium and placed in new cages to hatch.

Sample collection was performed using sterilised tools and a stereomicroscope, under CO_2_ anaesthesia. A biological triplicate of 25 pooled specimens of each developmental stage (eggs, larvae stage 1, 2 and 3, pre-pupa, pupa and adult male) were collected into Eppendorf tubes and stored immediately at −18 °C based on age and morphological characteristics^18^ from the F2 generation reared in the laboratory, taking care to not to transfer any media. Media samples were collected in Eppendorf tubes during the sampling of each life stage, near the collected specimens. Furthermore, the microbiome of ten individual male and female adult flies from F1 were evaluated, as well as available adult flies from F2 flies (10 male, 7 female). Finally, triplicate samples of 25 pooled adult male flies of the F2 generation were dissected into anatomical body parts (head, wings, legs, body, crop, midgut, hindgut) using sterilized tools and a stereo microscope. Briefly, legs and wings were removed under CO_2_ anaesthesia, followed by immediate removal of the head. The housefly gut was removed from the rest of the body, and transferred to a drop of sterilised demineralised water for sectioning into crop, mid- and hindgut. Tools were sterilised continuously between collection of the different fly compartments. Samples were transferred to 1.5 mL Eppendorf tubes and stored at −18 °C until further processing.

### DNA extraction and 16S rRNA gene amplicon sequencing

DNA extraction was performed in a dedicated clean work laboratory area, and all surfaces and non-disposable tools such as tissue grinders were sterilised using 70% ethanol and RNAse AWAY (Thermo Fisher Scientific) prior to use. Total DNA was extracted from all samples using the DNeasy Blood & Tissue kit (Qiagen, USA) according to the manufacturer’s protocol for extraction of DNA from insects with minor modifications. Tissues and media samples were subjected to a rough homogenization of using a tissue grinder (Thomas® Scientific) in 200 µL sterile PBS buffer prior to the addition of 180 µL ATL and 20 µL proteinase K (600 mAU·mL^−1^) and overnight incubation at 56 °C. Quality of extracted DNA was evaluated using Nanodrop 1000 (Thermo Fisher Scientific, USA) and 1% agarose gel electrophoresis. DNA quantity was assessed using Quant-IT dsDNA Broad Range assay kit (Thermo Fisher Scientific, USA) and TECAN infinite M1000 PRO plate reader (TECAN, Switzerland). The V1-3 variable region of the 16S rRNA gene was amplified using the primer set used by the Human Microbiome Project: 27F – AGAGTTTGATCCTGGCTCAG and 534R – ATTACCGCGGCTGCTGG^41^ fused with Illumina adaptors. Duplicate PCR reactions of 25 µL (1X Platinum High Fidelity Buffer, 2mU Platinum Taq DNA polymerase HF (Thermo Fisher Scientific, USA), 400 nM of each dNTP, 1.5 mM MgSO_4_, 400 nM of each primer and 10 ng of template DNA) were run under the following conditions: Initial denaturation at 95 °C for 2 minutes, followed by 30 cycles of denaturation at 95 °C for 20 seconds, annealing at 56 °C for 30 seconds and extension at 72 °C for 1 minute, and final elongation for 5 minutes at 72 °C. A negative control without DNA template and a sample of known content were included to control the quality of the amplicon generation. The generated amplicons were purified using AMPure XP bead protocol (Beckmann-Coulter, USA) using a sample:bead ratio of 0.8. Validation of the obtained libraries was performed using Qubit dsDNA High Sensitivity Assay kit (Thermo Fisher Scientific, USA), and TapeStation 2200 using D1000 ScreenTapes (Agilent, USA). Equimolar concentrations were sequenced using the Illumina MiSeq platform (Illumina, USA) using Illumina reagent kit v3 (2 × 300PE) with a 20% Phi-X spike-in.

Sequencing reads were quality checked and trimmed using trimmomatic (v0.32)^42^, merged using FLASH (v1.2.7)^43^ and formatted for use with the UPARSE workflow, which was used to exclude chimeric sequences^44^. Reads were then dereplicated and clustered into OTUs at 97% sequence similarity using USEARCH7^45^. Taxonomy was assigned using RDP classifier as implemented in QIIME with SILVA (release 132)^46^ as a reference database.

### Data analysis

Analysis of obtained data was performed using R version 3.5.2^47^ in RStudio version 1.0.463 (http://www.rstudio.com), using the R package ampvis2 (v2.19)^48^. Alpha diversity was calculated for all samples using observed OTU richness and evenness was calculated using the Shannon index^49^. Statistical testing of the observed alpha diversity between the individual life stages was performed using non-parametric Wilcoxon rank sum testing with Benjamini-Hochberg correction for multiple testing. Beta diversity was examined using Correspondence analysis (CA) on Hellinger transformed OTU abundances, as well as hierarchical clustering using the unweighted pair group method with arithmetic mean (UPGMA) method based on Bray-Curtis distances^50^ as calculated by the vegan package^51^. The microbial community structure was visualized using stacked bar plots and heatmaps based on relative abundance. Metabolic potential of the microbiota was predicted using Tax4Fun (version 0.3.1), as described elsewhere^37^, and subsequently analysed using STAMP^52^. All visualisations were generated with base R, and the packages ampvis2^48^ and ggplot2^53^.

## Supplementary information

Supplementary information.

## Data Availability

All sequence data used in this study has been made available at the European Nucleotide Archive (ENA) under project accession PRJEB31063. https://www.ebi.ac.uk/ena/data/view/PRJEB31063
