# Supplementary material for: Housefly (Musca domestica L.) associated microbiota across different life stages
Source: Sci Rep. 2020 May 12;10:7842. doi: 10.1038/s41598-020-64704-y (PMC7217826; doi:10.1038/s41598-020-64704-y)
Supplement: Supplementary file 1 — Supplementary information. [file 41598_2020_64704_MOESM1_ESM.docx]

**Supplementary Material to:**

**Housefly (*Musca domestica* L.) associated microbiota across different life stages**

Nadieh de Jonge, Thomas Yssing Michaelsen, Rasmus Ejbye-Ernst, Anne Jensen, Majken Elley Nielsen, Simon Bahrndorff, Jeppe Lund Nielsen*

*Department of Chemistry and Bioscience, Aalborg University, DK-9220 Aalborg East, Denmark*

**Keywords: housefly, life cycle, microbial community analysis, microbiome, life stage, ontogeny**

Corresponding author: jln@bio.aau.dk


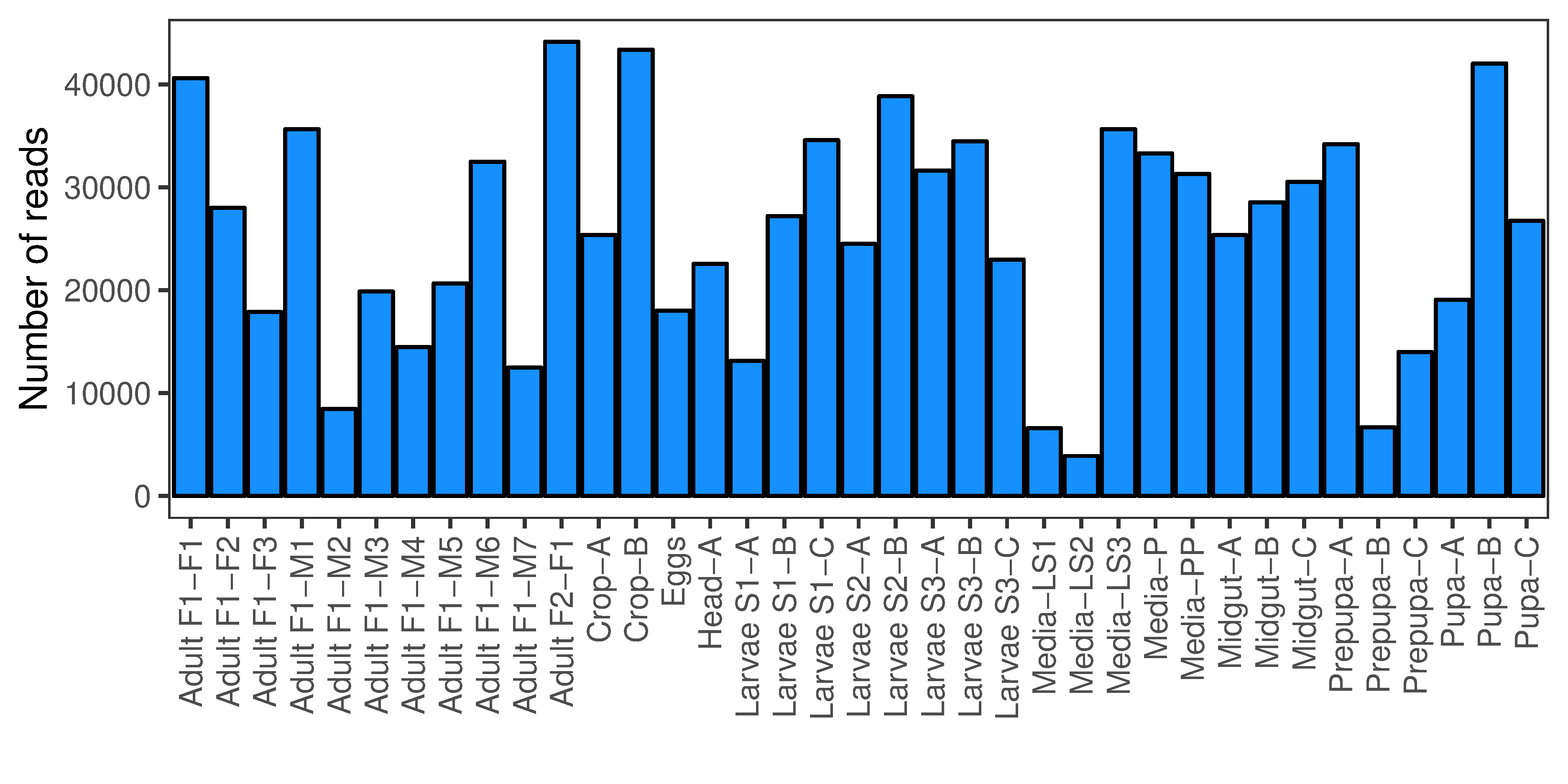


**Figure S1: Number of reads per sample.** Barplot of the number of high quality reads generated for in the individual samples included in the study. Samples are sorted alphabetically by name. Genders are abbreviated to F (female) and M (male), LS = Larval stage, P = pupa, PP = prepupa.


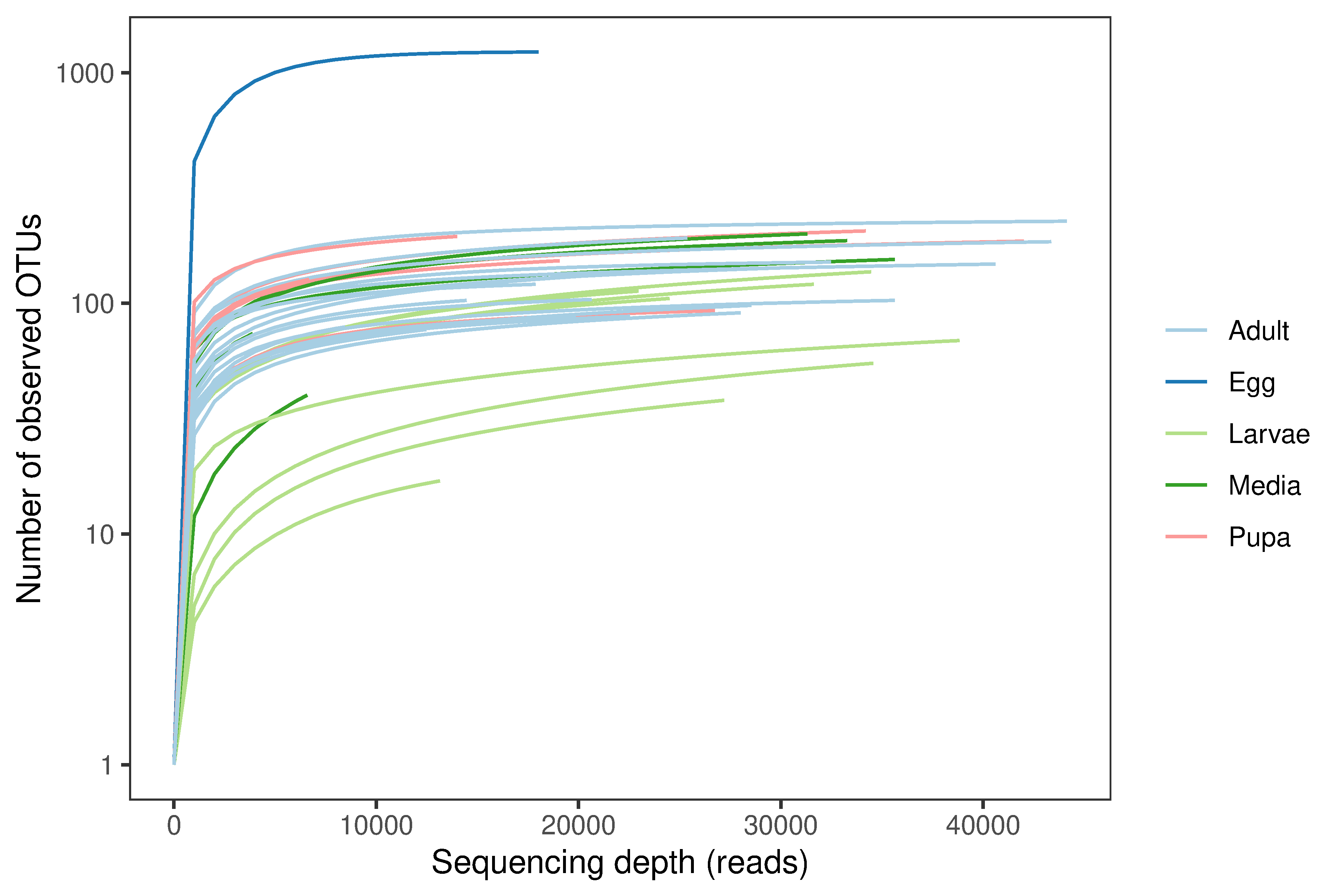


**Figure S2: Rarefaction curves of the individual samples.** Each individual curve represents one sample, and depicts the number of generated reads against the number of observed OTUs. Curves are coloured by overall sample type.


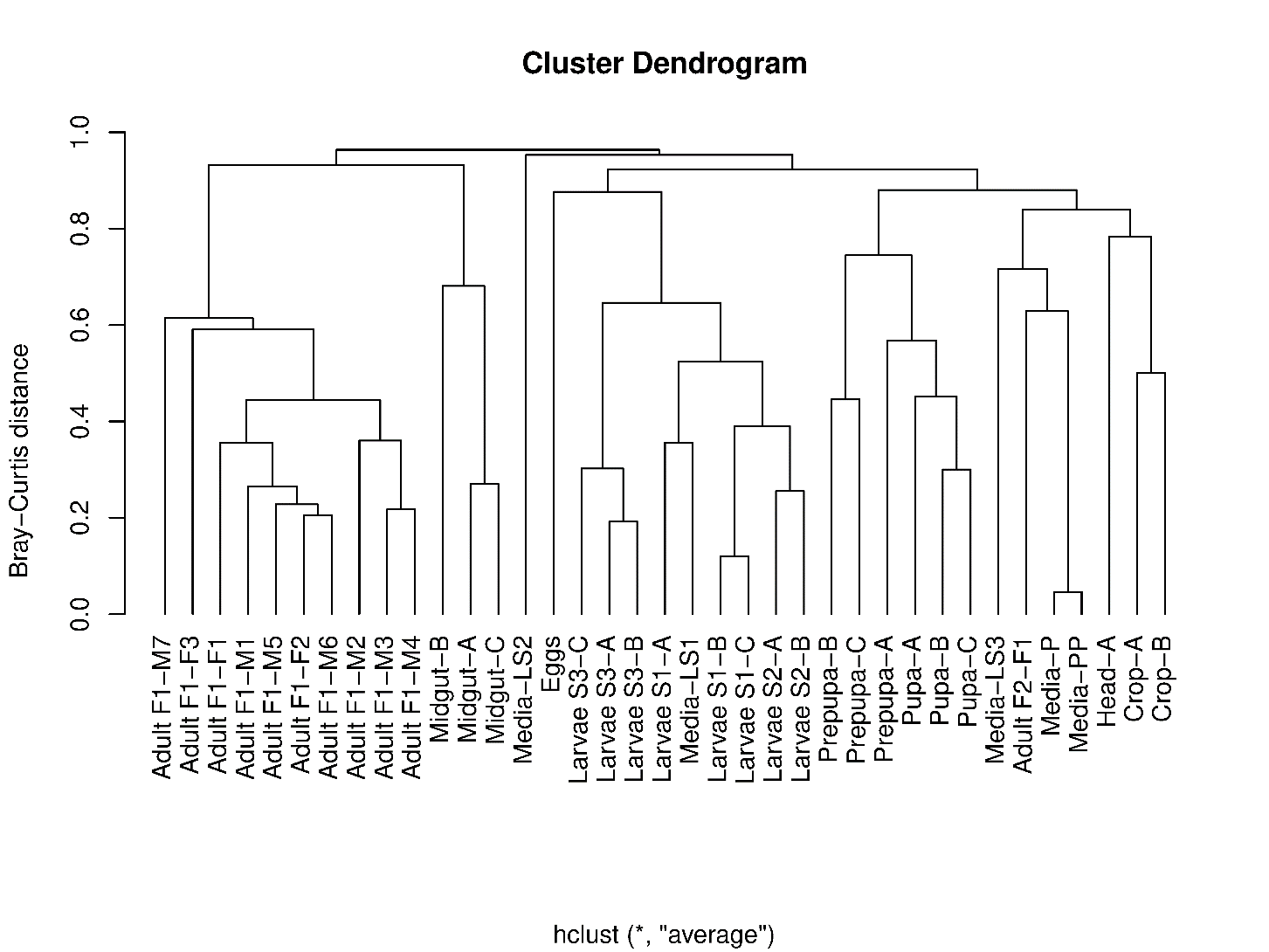


**Figure S3: Hierarchical clustering of the individual samples.** Hierarchically clustered dendrogram of Bray-Curtis distances between samples. Clustering was performed using the UPGMA method, and sample names are given. Genders are abbreviated to F (female) and M (male), LS = Larval stage, P = pupa, PP = prepupa.


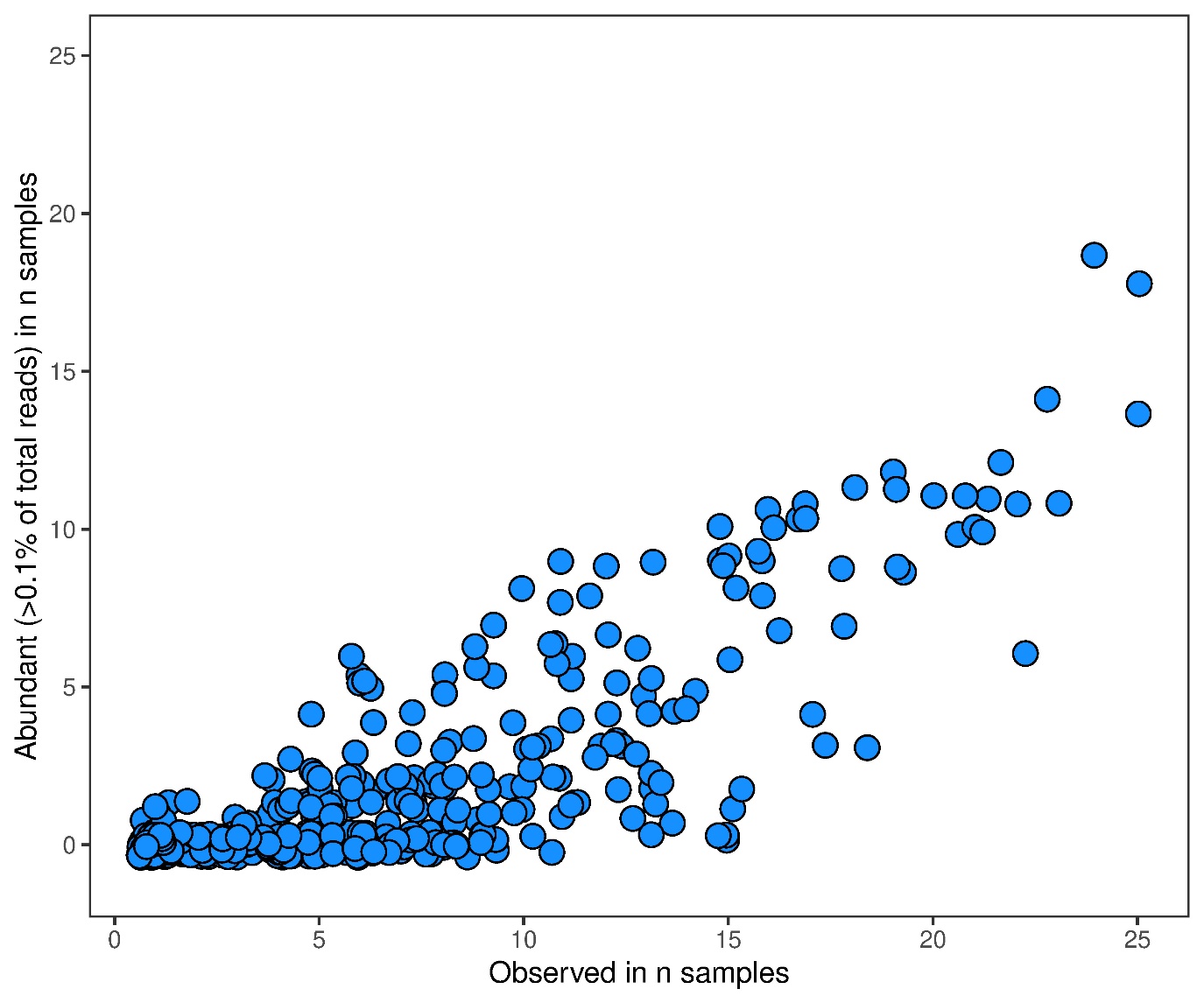


**Figure S4: Ubiquitously observed organisms across life stages.** Scatter plot of all OTUs depicting occurrence against abundant presence (> 0.1 % of total reads in a sample). All samples from the larval, pupal and adult life stages (n = 25) were included in the analysis.
